# Adolescent Attachment to Parents and Peers and the Use of Instagram: The Mediation Role of Psychopathological Risk

**DOI:** 10.3390/ijerph18083965

**Published:** 2021-04-09

**Authors:** Giulia Ballarotto, Barbara Volpi, Renata Tambelli

**Affiliations:** Department of Dynamic and Clinical Psychology, University of Rome “La Sapienza”, Via degli Apuli, 1, 00185 Rome, Italy; barbara.volpi@uniroma1.it (B.V.); renata.tambelli@uniroma1.it (R.T.)

**Keywords:** instagram addiction, social network addiction, adolescence, attachment to parents, attachment to peers, psychopathological risk

## Abstract

Several studies have shown an association between adolescents’ attachment relationships and social media use. Instagram is the social media platform most used by teenagers and recent studies have shown an association between Instagram use and increased psychopathological risk. The present study aims to verify whether psychopathological risk mediates the relationship between an adolescent’s attachment to their parents and peers and their Instagram addiction. N = 372 adolescents are assessed through self-report questionnaires evaluating Instagram addiction, the adolescents’ attachments to parents and peers, and their psychopathological risk. The Bergen Instagram Addiction Scale (BIAS) is developed by adapting the Bergen Social Media Addiction Scale. Results show the validity and reliability of the BIAS, confirming a one-factor structure. Findings show that a worse attachment to parents and peers is associated with adolescents’ psychopathological risk, which is associated with Instagram addiction. This finding has important clinical implications. Being able to intervene in adolescents’ relationships with parents and peers and the ways in which adolescents feel in relation to others could allow for a reduction in adolescents’ psychological difficulties, involving reduced Instagram use as a vehicle for the expression of their psychopathological symptoms.

## 1. Introduction

Adolescence is a period of life in which peers play an important role [1,2]. In this period, adolescents show a strong need to belong to a group [3] and social self-esteem is an important characteristic for the development of good emotional and behavioral functioning [4].

Social network platforms seem to meet these adolescents’ social needs [5]. One might expect that teenagers who frequently use social media have many social relationships that they feel satisfied with. However, several studies have not found this. Research has shown that adolescents who are more socially isolated and experience a greater sense of loneliness use social media more frequently [6], and that adolescents who show a stronger sense of belonging and have more positive relationships with their peers demonstrate a lower use of social networking platforms [7].

Therefore, it seems that the use of social network platforms leads to false need satisfaction. Indeed, a study by Scherr and Brunet [8] showed that young people with depressive symptoms published more status updates on Facebook than youths who showed no psychopathological symptoms. Furthermore, Sherlock and Wagstaff [9] found that increased use of Instagram was related to depressive symptoms, low self-esteem and body dissatisfaction, highlighting its possible contribution to psychopathological outcomes in adolescence.

On the other hand, Kircaburun and Griffiths [10] pointed out that youths who used Instagram in a problematic way used it as an escape from reality, suggesting that there was an individual vulnerability underlying the dysfunctional use of Instagram. Several studies have shown a relationship between attachment relationships and the use/abuse of social media [7,11], but while problematic social media use seems to be associated with increased psychopathological risk, research findings on social media addiction have been less thorough. The present study aims to investigate the complex relationship between relational factors (i.e., adolescents’ attachments to parents and peers), individual characteristics (such us the psychopathological risk) and social media addiction, in particular to Instagram.

### 1.1. Social Networkings Platforms and Instagram

Recent Digital 2020 data from We Are Social [12] showed that, in January 2020, 3.8 billion people were using social media, an increase of 9% from the previous year. The greatest users of social media today are youths. Indeed, social media offers several advantages to adolescents, such as allowing them to maintain their current friendships and form new ones [13], to easily find information and share their interests so that they can explore in a virtual space the possibilities of their evolving identity, as well as updating their profile and receiving feedback [14].

However, social media can also have some disadvantages. In fact, the quality of feedback plays an important role: positive feedback increases self-esteem and wellbeing, while negative feedback produces the opposite result in adolescents [4]. In addition, passive use (e.g., browsing other people’s profiles without publishing your own material) seems to be particularly harmful [15].

An interesting study [16] found that links between adolescents’ use of digital screens and their mental wellbeing do not follow a linear, but a curvilinear trend. This study highlighted how moderate use of digital technology is not intrinsically harmful, and may be advantageous in a connected world. In contrast, adolescents not using technology or using it excessively were found in the study to have worse mental wellbeing. Przybylski and Weinstein [16] looked at the use of different digital screens; while it is not a study specific to social media, the results are interesting: worse mental wellbeing was associated with problematic, excessive use of digital screens.

Recent literature shows that the reward system could be activated by specific aspects of the social media experience, such as likes, comments and follows [17,18]. Consequently, these features could give rise to addictive social media behaviors [19,20], including typical behavioral dependence behaviors [21], which are characterized by six specific components: conflict, withdrawal, salience, tolerance, mood modification and relapse [22]. In particular, studies seem to indicate that youths are particularly at risk of developing social media addiction [21,23].

Especially among youths, increasing in popularity are the highly visual social media platforms (HVSMs), which have gradually surpassed Facebook in popularity among adolescents [24,25]. HVSMs allow user-generated images or videos to be shared, with the possibility of using photographic filters to edit and improve the user’s appearance before uploading [26]. Among the HVSMs, the most popular and widely used platform is Instagram [12]. In Italy, Instagram is the 4th biggest social media application that is used, after YouTube, WhatsApp and Facebook, and the most used social media application among Italian teenagers [27], equally distributed by gender (50.5% females).

In addition to the use of visual content, the differences of Instagram from other social networking platforms, such as Facebook, concern reciprocity (unlike Facebook, where the connection with other users is mutual, following someone on Instagram is one-way) and the illusion of an always positive world; in fact, users are led to publish mainly positive situations, using various filters to enrich or embellish their content before publishing it.

An important study conducted in the United Kingdom [28] has shown that Instagram is the most harmful of all social media platforms for adolescents, highlighting the association of increased Instagram use with mental health problems such as sleep disorders, anxiety and depression. These results are supported by recent studies conducted in other cultural contexts, such as the United States [29], Italy [14], Peru [30], Iceland [15], France [31] and Spain [32], as well as intercultural samples [33,34].

Several authors have highlighted a causal effect in this association, pointing out that adolescents with psychopathological difficulties use Instagram in a dysfunctional way [10]. In fact, Verseillié and colleagues [31] found that depressive and anxious symptoms were predictive of problematic Facebook and Twitter use, and Jeri-Yabar and colleagues [30] highlighted how adolescents with pathological use of Instagram or Twitter had higher levels of depressive symptoms than adolescents with pathological use of Facebook, showing individual differences in the choice of pathological use of certain social networks. It is, therefore, necessary to assess the vulnerabilities of these adolescents (specifically to pathological use of Instagram). Several studies have also highlighted the importance of investigating relationships within the family context [35,36,37].

### 1.2. Attachments and Social Networks

Studies that have investigated the relationship between attachment and social media use have focused mainly on Facebook use [38,39,40]. Indeed, several studies have found an association between an insecure attachment and problematic Facebook use [38,41]. Furthermore, Rao and Madan [42] have found that adolescents with an insecure attachment show low confidence and a negative view of others and situations. In addition, they consider Facebook as a place to experience their independence, not suitable for the older generation. On the contrary, adolescents with secure attachment do not consider privacy and independence as big issues that revolve exclusively around Facebook use. They also appreciate the presence of their family members on Facebook.

Moreover, several studies have investigated the relationship between Facebook use and attachment, not only from a dichotomous perspective of security/insecurity, but also in terms of the quality of relationships between adolescents and their parents and peers [7,35,43]. Specifically, if on the one hand, Marino et al. [40] have found that attachment to the mother and father has an influence on problematic Facebook use, on the other hand, Badenes-Ribera and colleagues [7] have found that attachment to parents influences the abuse of Facebook in early adolescents, while during later adolescence it is more attachment to peers that influences its use. Furthermore, with regard to relationships with friends, Assunção and colleagues [43] have found that alienation from peers mediates the relationship between attachment to parents and problematic Facebook use.

When it comes to studies that have investigated the relationship between adolescents’ attachments and Instagram use, to our knowledge, only Ershad and Aghajani’s study [11] has done so. In particular, the authors found that neuroticism, alexithymia and insecure attachment (avoidant or ambivalent) were associated with Instagram addiction. On the other hand, numerous studies have shown an association between lesser communication and support within the family system and the emergence of problematic behaviors in adolescents [44,45,46]. However, no studies have investigated the relationship between adolescents’ attachments to their parents and peers and their Instagram addiction. Therefore, it seems that further studies are necessary to understand the implications of the use of this social media application.

### 1.3. The Present Study

In line with the Developmental Psychopathology theoretical framework [47,48,49,50,51] that considers clinical and subclinical forms of psychopathological problems (such as social networking addiction) to be the results of a complex interplay between relational risk factors (in particular, within the family context) and individual vulnerabilities, this study aims to understand how individual and relational characteristics contribute to the onset of Instagram addiction.

Farnicka and Bettin [52] analyzed the Developmental Psychopathology approach to diagnosing behavioral addictions in adolescence, highlighting that the analysis of determinants for addiction risk must consider many interrelated and complementary elements. In fact, the Developmental Psychopathology approach emphasizes how the pathways that follow healthy or disturbed development depend on the interaction of risk and protective factors throughout an individual’s life. Indeed, variability and instability in child and adolescent developmental pathways can result from positive (e.g., new peers) or negative changes in a youth’s environment. Therefore, to carefully analyze individual addictions, it is necessary to specifically address risk factors both understood as the factors responsible for susceptibility to addiction and the factors that directly initiate and sustain addictive behaviors [52]. During adolescence, the quality of the family and peer environment plays a significant role. In fact, parental influence decreases during this time, with a significant increase in the role of the peer group. The weakening of parental ties results in increased vulnerability to peer pressure, which can mediate the development of pathological behaviors [53].

A review from Lee, Ho and Lwin [54] analyzed the theoretical framework explicating the problematic use of social network sites, underlining the importance of considering relations with parents and peers as important elements that can influence adolescents’ problematic social network use. Among the theories that attribute addictive social media use to dispositional differences, attachment theory was cited the most [55,56]. Moreover, several studies stated that problematic behaviors online are more often a strategy to cope with psychological distress resulting from other psychopathological difficulties [57,58].

Based on these premises, this study aims to investigate the complex relationship between adolescents’ attachments to their parents and peers, psychopathological risk and Instagram addiction. Specifically, we hypothesize that individual psychopathological symptoms mediate the relationship between adolescent’s attachments to parents and peers and Instagram addiction.

As evidenced by the literature, the social network most used by adolescents is Instagram and its use is related to specific needs of adolescents (such as self-expression, social support and privacy from adults [59]), which are not met by other social networks such as Facebook. Consequently, in order to assess Instagram-specific addiction based on the six core features of addiction operationalized by Griffith [22], the Bergen Social Media Addiction Scale [60,61] questionnaire has been adapted to specifically investigate Instagram addiction. In fact, we believe that the specific characteristics of Instagram are different from those of other social networks, which were used by adolescents at the time of the development of the Bergen Social Media Addiction Scale. Therefore, we consider it necessary to adapt the scale and evaluate its psychometric properties.

## 2. Materials and Methods

### 2.1. Sample and Procedure

Through collaboration with high schools in Central-South and North Italy, N = 372 adolescents (42.2% boys and 57.8% girls) aged from 14 to 18 years (average age = 15.8; SD = 1.4) were recruited from April to June 2019. Most of the adolescents recruited for the study lived in families (99.5%). Of their parents, 83.9% were married or cohabiting (N = 312), while 11.3% (N = 42) were separated/divorced. With respect to parental educational level, 50.1% of fathers and 49.5% of mothers had high school degrees and 23.9% of fathers and 25.8% of mothers had graduated.

Before its start, this study was approved by the Ethical Committee of the Department of Dynamic and Clinical Psychology at Sapienza University of Rome, in accordance with the Declaration of Helsinki. Accordingly, an informed consent statement was signed by all adolescents and their parents. During school hours, psychologists and research assistants administered the self-report questionnaires (described below) to adolescents.

### 2.2. Measures

The Bergen Instagram Addiction Scale (BIAS) was developed by adapting the Bergen Social Media Addiction Scale [60,61], a 6-item self-report questionnaire developed to measure six core features of social media addiction: salience, mood modification, tolerance, withdrawal, conflict and relapse. Each item is scored on a five-point Likert scale from one (very rarely) to five (very often). Higher scores indicate greater Instagram addiction. Factor structure and psychometric properties are shown in the Results section.

The Inventory of Parent and Peer Attachment (IPPA; [62]) is a self-report questionnaire used to assess adolescents’ perceptions of their attachments to parents and peers. This measure assesses adolescents’ feelings of security and positive/negative aspects of the affective and cognitive dimensions featured in their relationship with parents and peers. It is composed of three parts relating respectively to the mother, father and friends. The parent sections are composed of 28 items for each parent, while the part relating to friends consists of 25 items. Each item is measured on a Likert five-point scale from one (never true) to five (always true). The Italian validation [63] showed good internal consistency, ranging from 0.62 to 0.90.

The Symptom Checklist-90-Revised (SCL-90-R [64]) is a self-report questionnaire composed of 90 items, evaluating participant’s psychopathological risk. Each item describes a physical or psychopathological symptom that the subject could have experienced in the last week, evaluating it on a Likert five-point scale from 0 (not at all) to 4 (very much). This tool provides a Global Severity Index (GSI), indicating the respondee’s total subjective distress. The Italian validation was carried out by Prunas and colleagues [65], who have demonstrated satisfactory internal consistency of the measure in Italian adolescents and adults (α coefficient, 0.70–0.96) with a clinical cut-off score ≥ 1 for the GSI indicating psychopathological risk [65]. In the following sections, we will refer to psychopathological risk by addressing the SCL-90-R score.

### 2.3. Data Analysis

Before performing data analysis, univariate normality was verified for all items of the BIAS, IPPA and SCL-90-R based on Kim’s [66] standard guidelines. Furthermore, univariate and multivariate outliers were identified; no case was removed.

Then, a confirmatory factor analysis (CFA) was performed to investigate a one-factor solution and to assess the construct validity of BIAS. The comparative fit index (CFI) and the root mean square error of approximation (RMSEA) were used as fit indices. With regard to CFI, as suggested by Bentler and Bonnett [67], values greater than or equal to 0.90 were accepted as indicators of good fit. Furthermore, as highlighted by Hu and Bentler [68], an RMSEA value lower than 0.06 is recommended.

Finally, Pearson’s product-moment correlation coefficient was conducted. After verifying the presence of significant correlations between the variables investigated, to test whether adolescents’ psychopathological risk mediated the relationship between adolescents’ attachments to parents and peers and Instagram addiction, a path analysis model was created. Standardized regression weights β indicated the strength of the linear relation and implied a direct relation between changes in the connected variables. Furthermore, to assess the overall fit of the data to the model, we considered chi-square values, goodness-of-fit indices and squared multiple correlations. The chi-square assessment of fit refers to the possibility for a hypothesized model to adequately fit the data. Goodness-of-fit indices range from zero to one, with values close to one indicating a good fit. Squared multiple correlations are indications of the amount of variability accounted for by the given equation. All statistical analyses were conducted with SPSS and AMOS.

## 3. Results

### 3.1. Descriptive Statistics

Table 1 shows the means and standard deviations of the main variables, and the straightforward correlations amongst them.

### 3.2. Factor Structure

The corrected item-total correlation coefficients for all six items are presented in the Supplementary Materials (see Table S1). For each of the six core addiction elements retained, the corrected item-total correlation coefficient ranged from 0.52 and 0.72. Furthermore, Cronbach’s alpha for BIAS was good (α = 0.795).

**Figure 1:**
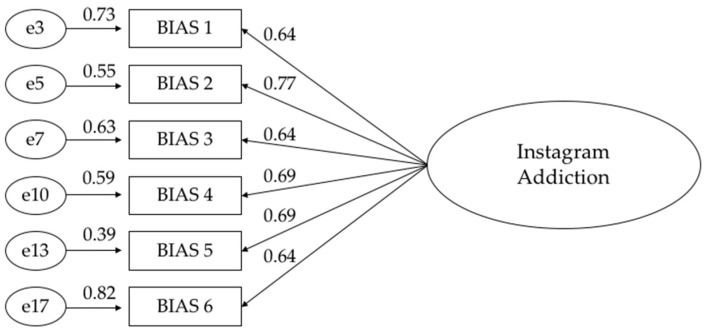
Factor structure and standardized loadings of items in the Bergen Instagram Addiction Scale (BIAS).

A CFA was performed on the six items of the BIAS, in order to test the pre-established one-factor solution of the Instagram addiction construct [10]. Results showed a one-factor structure (χ^2^/df = 0.672, *p* > 0.05) with good fit indices. Specifically, the root mean square error of approximation (RMSEA) of the model was 0.000, the comparative fit index (CFI) was 1.000, the normed fit index (NFI) was 1.000 and the Tucker-Lewis fit index (TLI) was 1.007. Overall, these results clearly demonstrate that the one-factor solution model presents an excellent fit to the data (Figure 1).

As shown in Figure 1, the standardized loadings of the six indicators of the one-factor solution in the BIAS ranged from 0.64 to 0.77.

### 3.3. Assessing the Mediating Role of Adolescents’ Psychopathological Risk

Pearson’s product-moment correlation coefficient was conducted to explore the associations between adolescents’ attachments to parents and peers, psychopathological risk and Instagram addiction. The results are shown in Tables S2 and S3.

After confirming the presence of significant correlations between variables, in order to investigate whether adolescents’ psychopathological risk mediates the relationship between adolescents’ attachments to parents and peers and Instagram addiction, a path analysis model was created. In the path model, adolescents’ attachments to mother, father, and peers covary among themselves. Furthermore, in order to evaluate indirect effects the bias-corrected bootstrap estimation method was used.

Conventional fit indices and thresholds were used to examine the goodness of fit of the model under analysis: χ^2^/df = 3.2 (*p* = 0.07) and the comparative fit index (CFI) was 0.99; the high level of CFI indicated the model’s good fit to the actual data. The resulting value for the root mean square error of approximation (RMSEA) was 0.07 and has been proposed to indicate excellent-to-acceptable fit. Furthermore, the normed fit index (NFI) was 0.99 and the Tucker-Lewis fit index (TLI) was 0.94. These results show that this solution model presents an excellent fit with our data.

**Figure 2:**
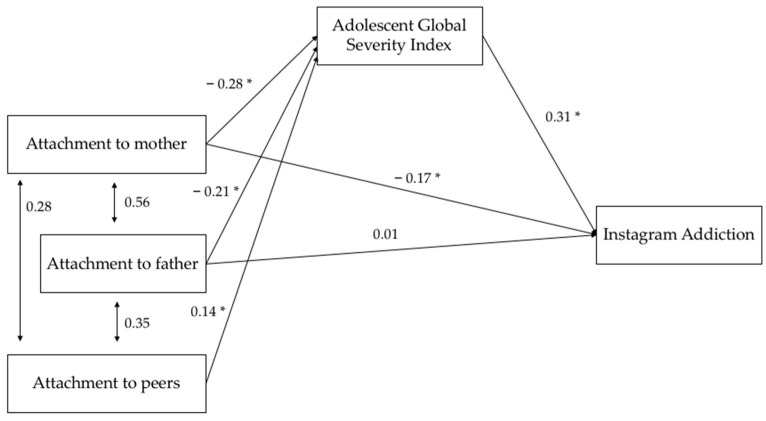
Path model standardized regression weights for the mediating role of adolescents’ Global Severity Index scores on the relationship between adolescents’ attachments to parents and peers and Instagram addiction (* *p* < 0.05).

Figure 2 gives the standardized structural parameter estimates for the model. The standardized total effects showed that Instagram addiction was influenced by attachment to the mother (β = −0.26; CI = [−0.39; −0.12]; *p* = 0.002) and peers (β = −0.04; CI = [−0.09; −0.01]; *p* = 0.007) but not by attachment to the father (β = −0.005; CI = [−0.2; 0.09]; *p* = 0.467). Specifically, adolescents’ Instagram addiction was directly influenced by attachment to the mother (β = −0.17; CI = [−0.29; −0.05]; *p* = 0.009) but not by attachment to the father (β = 0.01; CI = [−0.14; 0.14]; *p* = 0.924); on the other hand Instagram addiction was indirectly influenced by attachment to the mother (β = −0.09; CI = [−0.14; −0.05]; *p* = 0.001), father (β = −0.06; CI = [−0.13; −0.02]; *p* = 0.001) and peers (β = −0.04; CI = [−0.09; −0.01]; *p* = 0.007), through the mediating effect of adolescents’ scores on the Global Severity Index.

## 4. Discussion

The present study aimed to investigate the complex relationship between adolescents’ attachments to parents and peers, psychopathological risk and Instagram addiction.

In order to use a specific questionnaire for Instagram addiction assessment, based on the six core features of addiction operationalized by Griffith [22], a tool was developed from the Bergen Social Media Addiction Scale questionnaire [60,61]. The latter refers indiscriminately to different social media but, as highlighted by the literature [69], adolescents are driven to use different social media platforms to meet their different needs. Indeed, platforms such as Instagram better meet their needs for self-expression, social support and privacy from adults [59].

Firstly, the factorial structure and reliability of the Bergen Instagram Addiction Scale were assessed. Our hypothesis was that this tool had a unidimensional factor structure. Results confirmed our hypothesis and showed a one-facture structure with good fit indices (χ^2^/df = 0.672; RMSEA = 0.000; CFI = 1.000) and excellent internal consistency (α = 0.915). Furthermore, all items significantly loaded on the factor, with a range from 0.64 to 0.77. This result is consistent with previous studies. In fact, the Bergen Social Media Addiction Scale has been validated for several countries (e.g., Italy [61], Iran [70]) and has been adapted for Facebook addiction (Bergen Facebook Addiction Scale [71,72,73,74,75,76]). In all of these studies, it has shown a robust unidimensional factor structure. Our results show that the six core features of social media addiction identified by Griffith [22] (salience, mood modification, tolerance, withdrawal, conflict and relapse) were well evaluated by the BIAS, with a specific focus on Instagram addiction.

After confirming the reliability and validity of the BIAS, this study aimed to verify the relationships between the adolescents’ attachments to parents and peers, psychopathological risk and Instagram addiction.

Specifically, we hypothesized that the adolescents’ psychopathological risk mediated the relationship between their attachments to parents and peers and Instagram addiction. In particular, we aimed to verify that lower attachment to parents and peers increases psychopathological risk, and that the latter increases Instagram addiction.

The results confirmed our hypothesis. Specifically, the results showed that adolescents’ attachment to the mother predicted their Instagram addiction, both directly and indirectly (mediated by adolescents’ psychopathological risk). On the other hand, adolescents’ attachment to the father did not directly predict their Instagram addiction; this relationship was mediated by adolescents’ psychopathological risk. Furthermore, adolescents’ attachment to peers predicted their Instagram addiction only when mediated by adolescents’ psychopathological risk. These results highlighted the mediation role of adolescents’ psychopathological risk in the relationship between their attachments to parents and peers and their Instagram addiction. On the other hand, regarding adolescents’ relationship with the mother, it appears that this also plays a direct role in influencing increased teen Instagram addiction. However, it is important to note that the verified path model includes covariation among the variables of attachment to the mother, father and peers. In fact, it is hypothesized that there are additional unobserved variables that influence the quality of adolescents’ attachment. As evidenced by several studies, in line with the theoretical model of Developmental Psychopathology [47,48,49,50,51], developmental trajectories are complex and related to several individual (biological, temperamental) and environmental factors, which interact together [47,52]. Attachment relationships represent adolescents’ way of being with a significant other, necessarily linked to individual aspects; on the other hand, as also highlighted by the path model, adolescents’ ways of being with a significant other predict a problematic use of social networks, which, therefore, also concern a way of communicating with the other.

To our knowledge, this is the first study analyzing the mediation role of psychopathological risk in the relationship between adolescents’ attachments to parents and peers and their Instagram addiction. Studies on other social media addictions have found that attachment to the mother and father has an influence on problematic social media use [40]; the only study that specifically studied adolescents’ attachment and Instagram addiction [11] highlighted that avoidant and ambivalent attachment were associated with Instagram addiction, but this relationship has not been deepened.

As highlighted above, adolescents use Instagram to meet specific needs. In particular, Instagram is often used by teenagers to gain privacy from adults, who instead populate the world of Facebook. Furthermore, a need to express themselves and show positive images of themselves is greatly present among Instagram users. It is reasonable to think that adolescents with worse attachment relationships with their parents and peers may increasingly seek a social network away from the adult world (consistent with the study of Rao and Madan [42]), where they can seek greater social support and carry out identity-seeking behaviors by mirroring others.

The present study has several strengths. As already pointed out, this is the first study analyzing the mediation role of psychopathological risk in the relationship between adolescents’ attachment to parents and peers and their Instagram addiction. Furthermore, this study considers a large group of adolescents, homogeneous in gender and age. Finally, this study uses a specific measure to assess Instagram addiction, as was also recently performed in Ponnusamy and colleagues’ study [77].

Despite these strengths, this study has limitations. First, as evidenced by the covariation between adolescents’ attachments to parents and peers in the path model, and as discussed above, other constructs might be important in the relationship between adolescents’ attachments and Instagram addiction, such as adolescents’ emotional regulation [78,79] or epigenetic characteristics [80,81,82] or parents’ psychopathological risk [83,84], which have not been investigated.

Furthermore, although we used validated and frequently used measures, there could be a conceptual overlap between the Symptom Checklist and the BIAS subscales for Mood Modification and Withdrawal. On the other hand, the results shown in Table S3 highlighted that the correlations between the subscales for Mood Modification and Withdrawal with adolescent Global Severity Index were not higher than those between the latter and other BIAS subscales (*r* ranged between 0.21 and 0.28).

Future research should administer other measures to evaluate adolescents’ attachments, such as through adult attachment interviews [85]. Inevitably, this would, however, reduce the sample size and would not allow for the investigation of different attachment relationships (i.e., to parents and peers).

## 5. Conclusions

The digital revolution [86], which began at the end of the 1990s, entailed a structural change not only in the magical years of childhood [87], but also in the adolescent and adult world. For adults to responsibly fulfill their roles of protection and support, they must necessarily take into account the impact of new technological devices on the evolutionary process of childhood growth.

Social media is increasingly popular among young people and growing literature is evaluating its benefits, but also its risk factors. The literature has highlighted the link between social media abuse and increased psychopathological risk, but the nature of this relationship has been little explored.

Some authors have highlighted how there is an individual vulnerability underlying dysfunctional use of Instagram, the social media platform most used by teens.

Our study sought to understand more of the nature of this relationship, focusing on adolescents’ attachment. The results highlighted the mediating role of adolescents’ psychopathological risk, showing that a worse attachment to parents and peers was associated with adolescents’ psychopathological sufferance, which was associated with Instagram addiction. This finding has important clinical implications that may allow therapeutic and preventive interventions to be planned. In fact, being able to intervene in adolescents’ relationships with parents and peers, and in the ways that adolescents feel in relation to others, could allow a reduction in adolescents’ psychological difficulties, involving a reduction in Instagram use as a vehicle for the expression of their psychopathological symptoms.

On the other hand, it is important to take into account that our hypothesized relationship is only one possible one. Another might be that dysfunctional attachment leads to Instagram addiction, which leads to GSI. Further studies should further investigate this relationship.

## Figures and Tables

**Table 1:** Means, standard deviations and correlations of the main variables.

	Variables	Mean (SD)	1	2	3	4	5
1	Instagram addiction	32.59 (12)	1				
2	Global Severity Index	0.93 (0.6)	0.36 **	1			
3	Attachment to mother	47.41 (23.88)	−0.31 **	−0.44 **	1		
4	Attachment to father	54.72 (19.11)	−0.20 **	−0.43 **	0.56 **	1	
5	Attachment to peers	53.43 (13.72)	−0.07	−0.31 **	0.28 **	0.35 **	1

** *p* < 0.001.

## Data Availability

The data presented in this study are available on request from the corresponding author. The data are not publicly available for reasons of privacy.

## Supplementary Materials

The following are available online at https://www.mdpi.com/article/10.3390/ijerph18083965/s1, Table S1: The *Bergen Instagram Addiction Scale*: Items and Intercorrelations of Ratings; Table S2: Correlations between adolescent’s attachment to parents and peers and their psychopathological risk and Internet addiction; Table S3: Correlations between adolescent’s psychopathological risk and BIAS total score and items.
