# Supplementary material for: Adolescent Attachment to Parents and Peers and the Use of Instagram: The Mediation Role of Psychopathological Risk
Source: Int J Environ Res Public Health. 2021 Apr 9;18(8):3965. doi: 10.3390/ijerph18083965 (PMC8069955; doi:10.3390/ijerph18083965)
Supplement: Supplementary file 1 [file ijerph-18-03965-s001.pdf]

Supplementary Materials

Table S1. The Bergen Instagram Addiction Scale: Items and Intercorrelations of Ratings

	Item	Item-total correlation
**Salience**		
BIAS1	Spent a lot of time thinking about Instagram or planned use of Instagram?	0.524
**Tolerance**		
BIAS2	Felt an urge to use Instagram more and more?	0.723
**Mood modification**		
BIAS3	Used Instagram in order to forget about personal problems?	0.578
**Relapse**		
BIAS4	Tried to cut down on the use of Instagram without success?	0.607
**Withdrawal**		
BIAS5	Become restless or troubled if you have been prohibited from using Instagram?	0.649
**Conflict**		
BIAS6	Used Instagram so much that it has had a negative impact on your job/studies?	0.538

Table S2. Correlations between adolescent's attachment to parents and peers and their psychopathological risk and Internet addiction

	Adolescent Global Severity Index	Instagram Addiction
Attachment to mother	−0.43 **	−0.30 **
Attachment to father	−0.41 **	−0.21 **
Attachment to peers	−0.29 **	−0.06

**$p < 0.001$

Table S3. Correlations between adolescent's psychopathological risk and BIAS total score and items

	Adolescent Global Severity Index
Instagram Addiction (BIAS total score)	0.38 **
Salience	0.21 **
Tolerance	0.30 **
Mood Modification	0.37 **
Relapse	0.38 **
Withdrawal	0.25 **
Conflict	0.29 **

** $p < 0.001$
